# MiR-130b Is a Prognostic Marker and Inhibits Cell Proliferation and Invasion in Pancreatic Cancer through Targeting STAT3

**DOI:** 10.1371/journal.pone.0073803

**Published:** 2013-09-10

**Authors:** Gang Zhao, Jun-gang Zhang, Ying Shi, Qi Qin, Yang Liu, Bo Wang, Kui Tian, Shi-chang Deng, Xiang Li, Shuai Zhu, Qiong Gong, Yi Niu, Chun-you Wang

**Affiliations:** 1 Pancreatic Disease Institute, Union Hospital, Tongji Medical College, Huazhong University of Science and Technology, Wuhan, China; 2 Department of Hepatobiliary and Pancreatic Surgery, Zhejiang Provincial People’s Hospital, Hangzhou, China; 3 Department of Obstetrics and Gynecology, Zhejiang Provincial People’s Hospital, Hangzhou, China; Medical University Graz, Austria

## Abstract

Accumulating evidence indicates that microRNAs (miRNAs) are aberrantly expressed in human cancer and contribute to the tumorigenesis, but their roles in pancreatic cancer are still largely unknown. In this study, our data showed that miR-130b was significantly downregulated in 52 pairs of pancreatic cancer tissues and five cell lines. Furthermore, the deregulated miR-130b was correlated with worse prognosis, increased tumor size, late TNM stage, lymphatic invasion and distant metastasis. Multivariate analysis showed that miR-130b expression was a significant and independent prognostic predictor for pancreatic cancer patients. Functional studies indicated that the overexpression of miR-130b dramatically suppressed the proliferation of pancreatic cancer cells both in vitro and in vivo, which could be attributed to the induction of apoptosis and cell cycle arrest at S phase. Meanwhile, an overexpressed miR-130b remarkably inhibited the invasive ability of pancreatic cancer cells. Moreover, the dual luciferase assay revealed that STAT3 was directly targeted by miR-130b, which was further confirmed by the inverse expression of miR-130b and STAT3 in pancreatic cancer samples. Our findings suggested that miR-130b might have a considerable potential in prognosis identification and application of therapy for pancreatic cancer.

## Introduction

MicroRNAs are endogenous non-coding RNAs consisting of about 22 nucleotides. MicroRNAs can negatively regulate gene expression by binding to partially complementary sequences in the specific target mRNA 3′-untranslated region (UTR), which can result in either mRNA degradation or translation inhibition [1]. Emerging evidence indicates that miRNAs are aberrantly expressed in different types of tumors and participate in human tumorigenesis and/or metastasis by directly targeting oncogenes or tumor suppressor genes [2]–[4].

Pancreatic cancer is one of the most lethal malignancies. Only 10–20% of the patients diagnosed with pancreatic cancer are resectable and overall its 5-year survival rate is only 5% due to its high recurrence rate despite the multimodality treatments [5], [6]. Like other cancers, the development of pancreatic cancer is a multistep process with accumulation of genetic and epigenetic changes. Altered miRNA expressions, such as miR-34a, miR-21 and miR-20a, have been identified as modulators of cell growth, apoptosis, migration or invasion in pancreatic cancer [7]–[9]. Therefore, more extensive investigations are needed on the role of miRNAs, which are deregulated in pancreatic cancer in order to elucidate the function of miRNAs in pancreatic cancer.

Microarray studies have identified a number of microRNAs that were deregulated in pancreatic cancer, including miRNA-130b [10]–[12]. To date, miR-130b has been found to be deregulated in some types of cancers including being overexpressed in gastric cancer [13], [14], glioma [15] and renal cell cancer [16], while being downregulated in endometrial cancer [17] and papillary thyroid carcinoma [18]. However, no specific studies have been conducted to reveal the role of miR-130b in pancreatic cancer. Hence, our study was aimed to identify the role of miR-130b in pancreatic cancer. In present study, the expression of miR-130b between pancreatic cancer and the normal adjacent pancreatic tissues were analyzed. Furthermore, the proliferation and invasiveness were evaluated in PANC-1 and ASPC-1 cells after being transfected with miR-130b.

Our research further identified the potential direct target by which the miR-130b exerted its function on pancreatic cancer cells. The microRNA prediction software indicated that the signal transducer and activator of transcription 3 (STAT3) might be the downstream target of miR-130b. STAT3 is a key cytoplasmic transcription factor that is activated by tyrosine kinase growth and cytokine receptors. STAT3 plays an important role in mediating various biological processes including: cell proliferation, apoptosis and differentiation [19]. STAT3 has been identified as a key oncogenic factor in a number of malignancies and is required for oncogenesis in the skin and gastric cancers [20], [21]. In pancreatic cancer, activation of STAT3 promoted tumor cell growth and invasion, which led to poor patient survival [22]. Furthermore, STAT3 knockdown inhibited the cell growth and invasiveness in pancreatic cancer both *in vitro* and *in vivo*, and markedly decreased VEGF and MMP-2 expressions [23], [24]. Therefore, the dual luciferase assay was applied to identify whether STAT3 was directly targeted by miR-130b. Furthermore, the correlation between the expression of miR-130b and STAT3 in pancreatic cancer samples was further explored.

## Materials and Methods

### Patients and Tumor Tissues

A total of 52 human pancreatic cancer tissues (PC) and matched normal adjacent pancreatic tissues (NP) were obtained during the surgery at Pancreatic Disease Institute, Union Hospital (Wuhan, China) between January 2007 and December 2009. The diagnosis was based on pathological evidence and the specimens were immediately snap-frozen. They were stored at −80°C for future miR-130b and STAT3 extraction. None of the patients received chemotherapy or radiotherapy before the surgical excision. All 52 patients provided written informed consent for the use of their tissues and the study protocol was approved by the Ethics Committee of Huazhong University of Science and Technology, and the number of ethical approval was S214.

### Quantitative Real-time Reverse Transcription-polymerase Chain Reaction (qRT-PCR)

Total RNA was isolated from pancreatic cancer tissues or cells using Trizol reagent (Invitrogen, Carlsbad, USA). MiR-130b and U6 were polyadenylated using poly-A polymerase based First-Strand Synthesis kit (TaKaRa Bio, Japan) following the manufacturer’s protocol. To quantify the STAT3 and GAPDH mRNA levels, 1 ug of total RNA was subjected to first-strand cDNA synthesis for 15 min at 37°C and 5 s at 85°C using a PrimeScript RT Reagent kit (TaKaRa). The qPCR was performed using SYBR Green PCR master mix (TaKaRa) on the ABI 7500HT System. The U6 or GAPDH were used as an endogenous control. The relative fold expressions were calculated with the 2^−ΔΔCT^ method. All the qRT-PCR reactions were run in triplicate. A list of primers used in the reactions is presented in Table S1.

### Cell Lines and Cultures

Human pancreatic cancer PANC-1, ASPC-1, Miapaca-2, BXPC-3 and SW1990 cell lines were obtained from American Type Culture Collection (ATCC, Manassas, VA, USA) and were maintained in RPMI-1640 medium supplemented with 10% fetal bovine serum and antibiotics (100 U/ml penicillin and 100 µg/ml streptomycin sulfate). The cells were grown in a humidified incubator at 37°C with 5% CO2.

### Transfection

The microRNAs were designed and synthesized by RiboBio (Ribobio Co., Guangzhou, China). The microRNA transfection was performed using Lipofectamine 2000 (Invitrogen). The pancreatic cancer cells were seeded in 12-well plates and were grown up to 40% confluence before the transfection. The RNA and proteins were extracted at 48 h after the transfection. The final concentration of miR-130b mimic and anti-miR-130b was 50 nM. Lentiviral miR-130b (LV-miR-130b) and empty lentiviral vector (LV-NC) were constructed by Genechem Company (Shanghai, China) and were transfected into the pancreatic cancer cells according to the manufacturer’s instruction. All the oligonucleotide sequences used in this experiment are listed in Table S2.

### Cell Proliferation Assays

Cell proliferation was determined by MTT assays. Briefly, the pancreatic cancer cells (5×10^3^ per well) were plated in 96-well plates in RPMI 1640 and 10% FBS. After 24 h of being in the culture, the cells were transfected with 50 nM miR-130b mimics, anti-miR-130b and their respective control using Lipofectamine 2000 (Invitrogen). The cells were then cultured in the medium for another 48 h and were assessed by a colorimetric assay using MTT solution (5 mg/mL) at 570 nm. All the experiments were performed three times with five replicates.

### Evaluation of Apoptosis and Cell Cycle Distribution

Apoptosis rate and cell cycle stage were determined by FACS flow cytometry as previously reported [25]. For apoptosis analysis, cells were collected, washed twice with cold phosphate buffered saline (PBS) and resuspended in binding buffer at a cell density of 1×10^6^/mL. Cells were then stained with Annexin V-FITC and propodium iodide according to the manufacturer’s protocol. The signal was acquired by a FACS Calibur flow cytometer (BD Biosciences) and was analyzed with Cellquest software. For the cell cycle analysis, cells were harvested by trypsinization, washed twice using cold PBS and fixed in 70% ethanol overnight at −20°C. Then cells were treated with DNA staining solution containing 3.4 mM Tris-Cl (pH 7.4), propodium iodide, 0.1% triton X-100 buffer and 100 µg/ml RNase A. Cell cycle analysis was performed with FACS flow cytometry.

### Matrigel Invasion Assay

The Matrigel invasion chamber was used to assess cell invasion ability (24-well plates, 8 mm pore size, Corning) as previously described [26]. In brief, cells (5×10^4^) were seeded in the upper chamber at 37°C with the media containing 0.1% bovine serum albumin, while the media containing 20% fetal bovine serum was placed in the lower well. After 48 hours, the noninvading cells were removed with cotton swabs. Invasive cells at the bottom of the membrane were stained with 0.1% crystal violet and were counted under microscopic observation. The assays were performed in triplicate and were repeated three times.

### Western Blot Analysis

Pancreatic cancer cells were collected after 48 h treatment with 50 nM miR-130b mimic or anti-miR-130b and corresponding controls. Protein extraction, SDS-PAGE gel electrophoresis and blotting were performed as we previously described [27]. Several different primary antibodies were used including: STAT3 (Cell Signaling Technology, Danvers, USA) and GAPDH (Santa Cruz Biotechnology, Santa Cruz, USA). The secondary antibody incubations were performed for 2 h at room temperature and protein bands were visualized on the X-ray film using an enhanced chemiluminescence ECL substrate.

### Dual Luciferase Assay

Co-transfection experiments were performed in 96-well plates. A total of 1×10^4^ cells were seeded per well in 200 µl medium. A total of 100 ng wild type (WT) or mutant (MUT) reporter constructs were co-transfected with Lipofectamine 2000 transfection reagent into the pancreatic cancer cells with 50 nM miR-130b or miR-NC according to the manufacturer’s instruction. After 48 h, luciferase activity was measured with the Dual-Luciferase reporter assay system (Promega). Firefly luciferase activity was then normalized to the corresponding Renilla luciferase activity.

### Pancreatic Cancer Xenograft Model

Four-week-old female nude mice (BALB/c-nude) were used to examine the tumorigenicity. A total of 100 uL cell solutions (containing 1.5×10^7^ PANC-1 cells) were subcutaneously injected into the right flank of the mice. The tumor size was measured with a vernier caliper every four days and the tumor volumes were calculated using the formula: length×width^2^×0.5 [28]. The use of nude mice complied with the Guide for the Care and Use of Laboratory Animals and the study was approved by Animal Care and Use Committee of Tongji Medical College of Huazhong University of Science and Technology.

### Statistical Analysis

The miR-130b expression was compared in pancreatic cancer tissues and cells by the unpaired Student’s t test. The relationships between miR-130b and clinicopathologic parameters were evaluated by χ^2^ test. The survival rates for miR-130b expression were estimated by using the Kaplan–Meier method and the difference in survival curves were tested by log-rank test. The survival data were evaluated using a multivariate Cox regression analysis. The relationship between miR-130b and STAT3 expression was explored by Spearman’s correlation. All statistical analyses were carried out by SPSS13.0 software and the data were presented as means±standard deviation (SD). A p lower than 0.05 was considered significant.

## Results

### Clinicopathologic Significance of miR-130b in Pancreatic Cancer Patients

**Figure 1:**
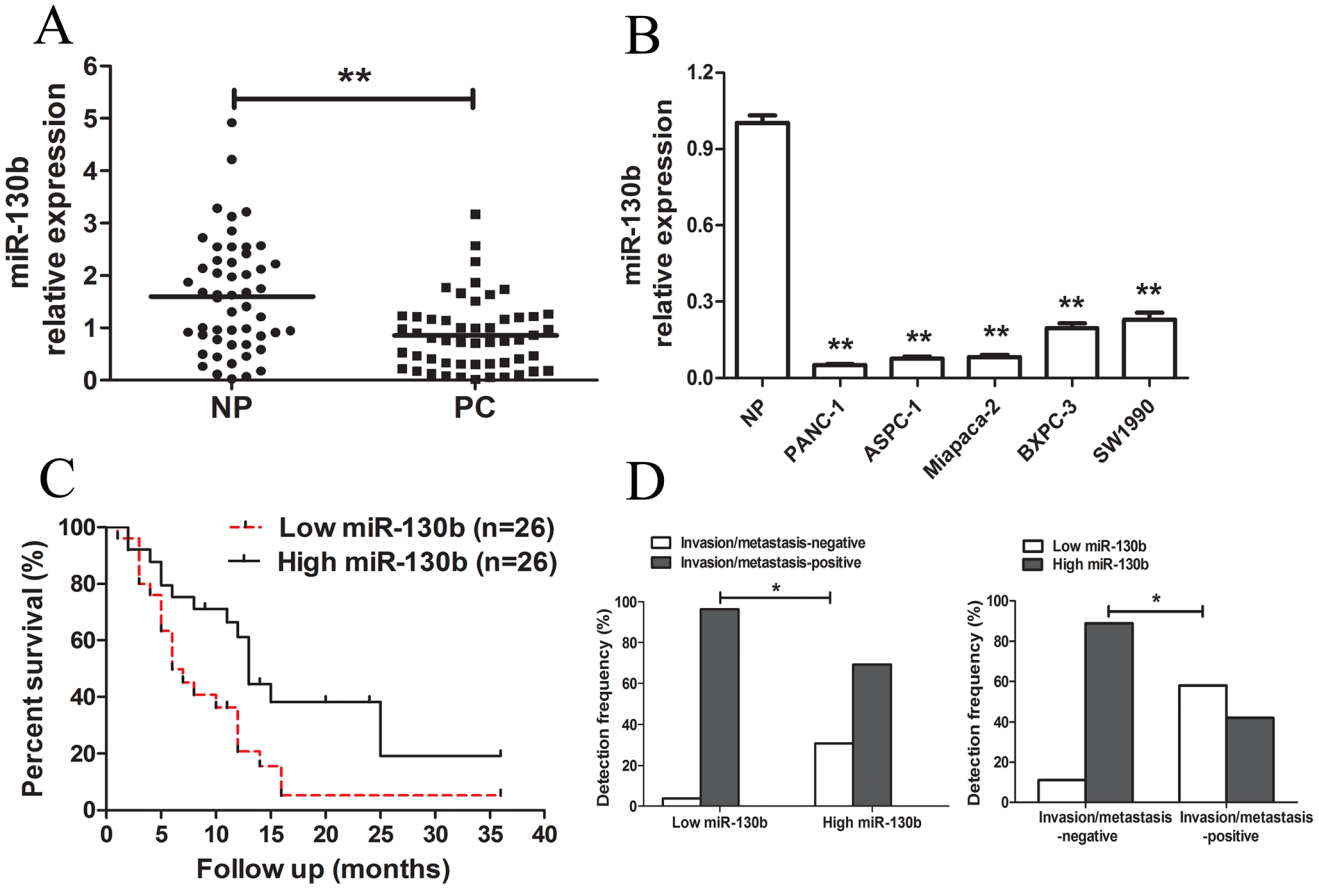
Analysis of miR-130b expression in human pancreatic cancer tissues and cell lines, and the pancreatic cancer patient survival. (A) The relative expression level of miR-130b in human pancreatic cancer tissues (n = 52) and matched adjacent noncancerous pancreatic tissues (n = 52). PC: pancreatic cancer tissues; NP: adjacent noncancerous pancreatic tissues. (B) The expression level of miR-130b in five pancreatic cancer cell lines (PANC-1, ASPC-1, Miapaca-2, BXPC-3 and SW1990). Data are presented in pancreatic cancer cell lines relative to the control. There was no normal pancreatic cell line, so we randomly chose three normal pancreatic tissues as control. U6 was used as the control for RNA loading and miRNA abundance was normalized to that of the U6 RNA. (C) Kaplan-Meier curves of the overall survivals of 52 pancreatic cancer patients were scored as low expression level (below the median value, n = 26) and high expression level (above the median value, n = 26) according to the miR-130b expression. The miR-130b downregulation was significantly correlated with an overall shorter survival. The P-values are shown with the use of log-rank test. (D) The χ^2^ analysis of relationship between miR-130b and invasion/metastasis in pancreatic cancer patients. *. P<0.05; **. P<0.01.

Using a qRT-PCR method, miR-130b was detected in all the 52 pairs of pancreatic cancer tissues and their matched noncancerous pancreatic tissues, as well as pancreatic cancer cell lines. As shown in Fig. 1A, 45 PC tissues showed low expression of miR-130b as compared to that of the NP tissues and the median fold change was 1.86 (P<0.01). Meanwhile, the miR-130b expression was significantly decreased in all five pancreatic cancer cell lines examined as compared to that of the normal pancreatic samples (Fig. 1B).

Furthermore, the correlation of miR-130b downregulation correlated with pancreatic cancer prognosis was investigated. We then studied the correlation between miR-130b expression and clinical pathological characteristics of pancreatic cancer. The low miR-130b expression group showed a higher incidence of an increased tumor size (P = 0.001), late TNM stage (P = 0.005), lymphatic invasion (P = 0.012) and distant metastasis (P = 0.012). However, no significant differences were observed with respect to sex, age, tumor location, histologic grade or vessel infiltration in pancreatic cancer (Table 1). Moreover, the Kapan-meier survival analysis revealed that the patients with a low miR-130b expression had a significantly poorer prognosis than those with a high expression (Fig. 1C). As shown in Fig. 1D, the χ^2^ analysis showed that patients with a lower miR-130b expression were more frequently associated with tumor invasion and metastasis. Furthermore, patients with tumor invasion and metastasis had a significantly lower miR-130b expression. Meanwhile, Cox’s multivariate analysis showed that miR-130b expression, TNM stage, and distant metastasis were significantly associated with overall survival of pancreatic cancer patients as independent prognostic factors (Table 2). These results showed that the miR-130 deregulation was correlated with a worse prognosis and was involved in invasion/metastasis of pancreatic cancer.

**Table 1:** The relationship between miR-130b and clinical pathological characteristics in 52 patients with pancreatic cancer.

		miR-130b expression		
Parameters	Numberof cases	High	Low	*P* value
**Sex**				
Male	34	18	16	0.56
Female	18	8	10	
**Age (y)**				
<60	31	16	15	0.777
≥60	21	10	11	
**Location**				
Head	32	15	17	0.826
Other	20	10	10	
**Tumor Size (cm)**				
<2	15	13	2	0.001*
≥2	37	13	24	
**Histologic grade**				
High/moderate	23	13	10	0.182
Poor	29	11	18	
**TNM stage**				
I∼II	24	17	7	0.005*
III∼IV	28	9	19	
**Lymphatic invasion**				
Positive	28	10	18	0.012*
Negative	24	17	7	
**Vascular invasion**				
Positive	27	12	15	0.405
Negative	25	14	11	
**Distant metastasis**				
Positive	24	7	16	0.012*
Negative	28	19	10	

* Statistically significant (P<0.05).

**Table 2:** Univariate and multivariate analysis of factors associated with overall survival in pancreatic cancer patients.

Characteristics	Univariate analysis		Multivariate analysis	
	HR (95% CI)	*P* value	HR (95% CI)	*P* value
Sex	1.389(0.482–3.999)	0.543		
Age	0.982(0.931–1.036)	0.511		
Tumor Size	0.636(0.197–2.056)	0.45		
histologic grade	0.763(0.485–1.201)	0.243		
TNM stage	6.107(1.386–26.914)	0.017*	2.602(1.037–6.530)	0.042*
Lymphatic invasion	0.715(0.185–2.762)	0.626		
Vascular invasion	0.681(0.292–1.588)	0.373		
Distant metastasis	8.358(2.127–32.845)	0.002*	5.819(2.149–15.754)	0.001*
miR-130b	0.291(0.123–0.688)	0.005*	0.352(0.155–0.797)	0.012*

HR, hazard ratio; CI, confidence interval;

* Statistically significant (P<0.05).

### MiR-130b Suppressed Cell Proliferation both *in* *vitro* and *in vivo*

**Figure 2:**
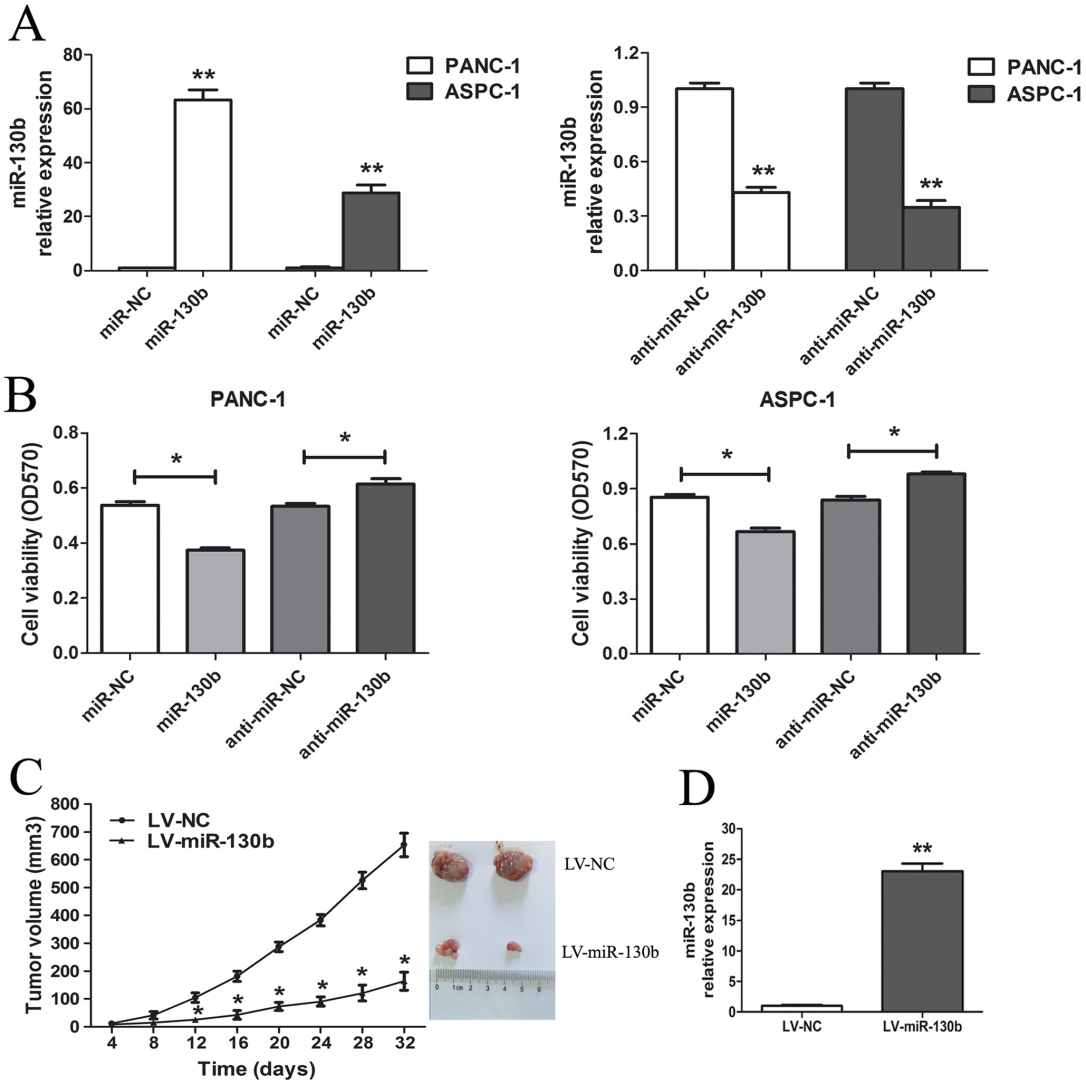
MiR-130b inhibited the growth of pancreatic cancer cells *in vitro* and *in vivo*. (A) The expression level of miR-130b was tested for 48 h in pancreatic cancer cells transfected with miR-130b mimics (miR-130b), anti-miR-130b and their respective NCs (50 nM) by qRT-PCR. (B) The effect of transient transfection of miR-130b or anti-miR-130b (50 nM) for 48 h was examined on the growth of PANC-1 and ASPC-1 cells by MTT assay. (C) The miR-130b inhibited the tumourigenicity in the nude mice xenograft model. The LV-miR-130b or LV-NC was transfected into PANC-1 cells in the presence of the virus at a multiplicity of infection (MOI) of 20. The infected PANC-1 cells were then subcutaneously injected into the nude mice. The tumor growth curves and the photographs of the excised tumors at 32 days post implantation are shown as indicated. Data are represented as the mean±SD in 10 mice. (D) The miR-130b expression levels were analyzed in excised tumors by qRT-PCR and were normalized to that of the endogenous control (U6 RNA). Data are shown as the mean±SD in 10 mice. *. P<0.05; **. P<0.01 as compared to the control.

In order to identify the effects of miR-130b on pancreatic cancer cells, we transfected PANC-1 and ASPC-1 cells with 50 nM miR-130b mimics, anti-miR-130b and their respective NCs. As shown in Fig. 2A, the miR-130b mimics caused a 63.32 and 28.75-fold increase in the miR-130b expression in PANC-1 and ASPC-1 cells, respectively. Meanwhile, the anti-miR-130b decreased the miR-130b expression by 2.34 and 2.88 folds in PANC-1 and ASPC-1 cells, respectively. After transfection with miR-130b mimics, the proliferation was significantly inhibited in PANC-1 and ASPC-1 cells by 30.35±2.90% and 22.03±7.64%, respectively (P<0.01). However, anti-miR-130b promoted the growth of PANC-1 and ASPC-1 cells by 15.23±7.40% and 16.70±3.56%, respectively (P<0.01; Fig. 2B).

To further confirm the above findings, an *in vivo* tumor model was constructed by implanting the PANC-1 cells transfected with LV-miRNA-130b or negative control. Throughout the tumorigenic period, tumors from the miR-130b transfected cells grew significantly slower (Fig. 2C). The miR-130b expression in xenograft tumors of LV-miR-130b group was obviously higher than that of the control group (Fig. 2D). These results demonstrated that miR-130b inhibited the proliferation of pancreatic cancer cells both *in vitro* and *in vivo*.

### MiR-130b Induced Apoptosis and Cell Cycle Arrest in Pancreatic Cancer Cells

**Figure 3:**
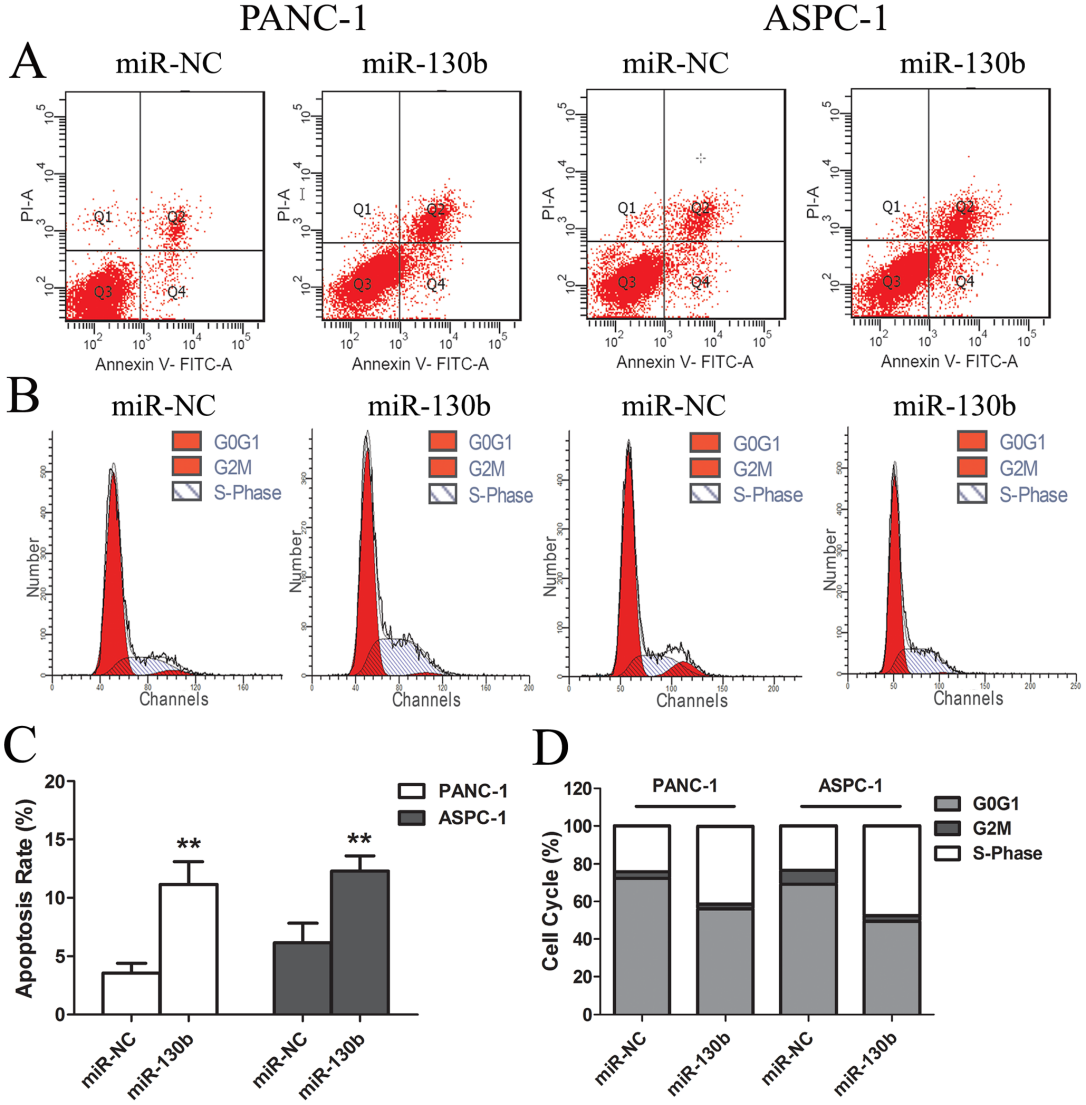
MiR-130b induced the apoptosis and cell cycle arrest in the pancreatic cancer cells. (A) PANC-1 and ASPC-1 cells were transfected with miR-130b or miR-NC (50 nM) for 48h and the apoptosis was measured by Annexin V staining and flow cytometry. (B) The PANC-1 and ASPC-1 cells were treated as mentioned above and the cell cycle distribution was measured by PI staining and flow cytometry. Flow cytometric analysis of the effects of miR-130b induced apoptosis (C) and cell cycle arrest (D). Data are presented as mean±SD of results from 3 independent experiments. **. P<0.01 as compared to the control.

To investigate the mechanisms of miR-130b inhibition on pancreatic cancer cell proliferation, we analyzed the apoptosis and cell cycle in the transfected PANC-1 and ASPC-1 cells using flow cytometry. The miR-130b mimics significantly increased the apoptosis rate in PANC-1 (3.57±0.83% *vs.* 11.17±1.96%) and ASPC-1 cells (6.17±1.66% *vs.* 12.30±1.30%) as compared to that of the miR-NC (Fig. 3A and 3C). Moreover, the miR-130b mimics significantly reduced the proportion of G0/G1 and G2/M phases. Furthermore, they increased the proportion of S phase in PANC-1 (24.13±4.10% *vs.* 41.54±3.80%) and ASPC-1 cells (23.52±3.33% *vs.* 47.66±2.04%) as compared to that of the controls (Fig. 3B and 3D).

### MiR-130b Inhibited the Pancreatic Cancer Cell Invasiveness

**Figure 4:**
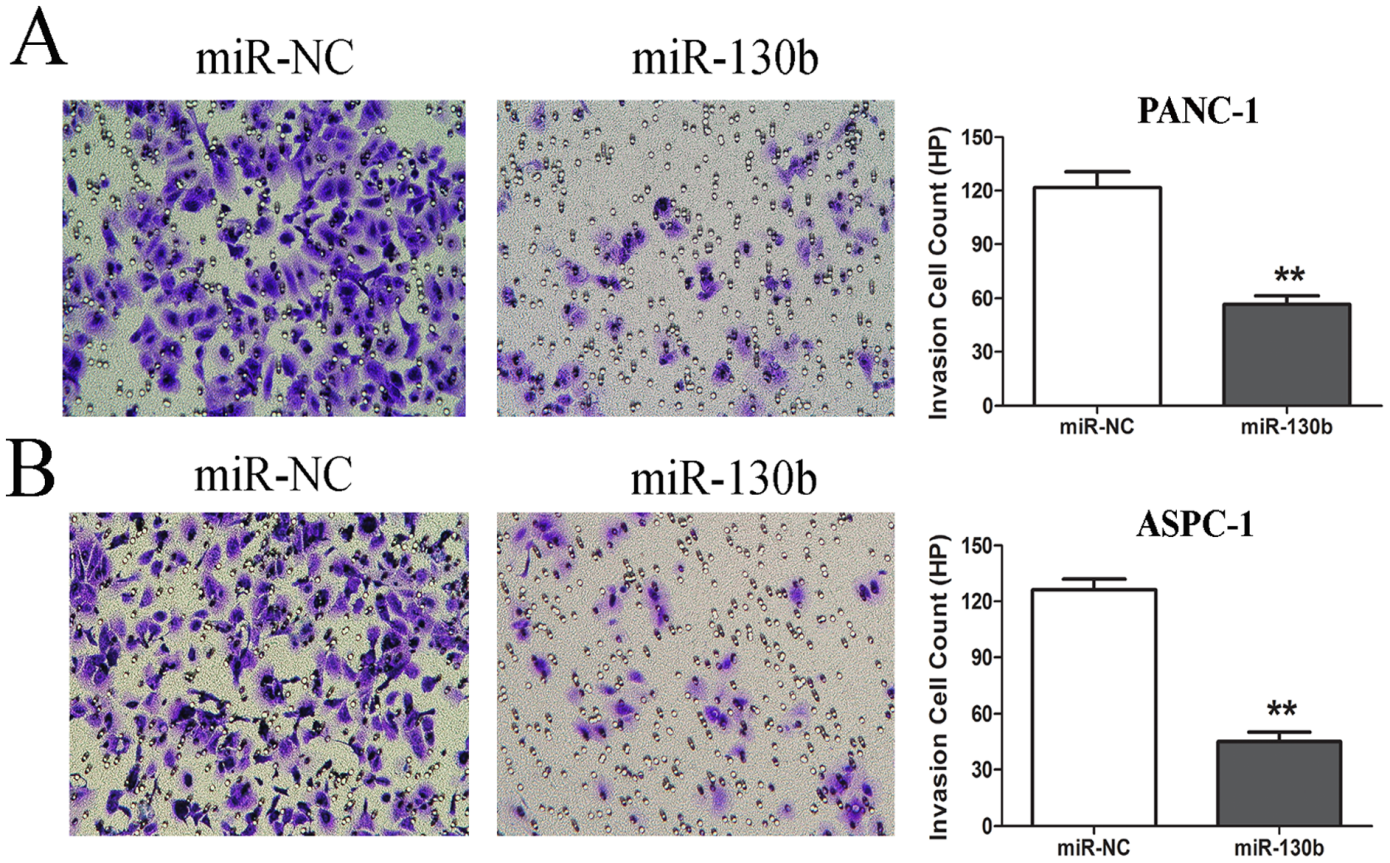
MiR-130b inhibited the invasion of pancreatic cancer cells. (A) The PANC-1 cells were transfected with miR-130b or miR-NC (50 nM) for 48h and the cell invasion ability was measured by matrigel invasion assays. (B) The ASPC-1 cells were treated as above and the cell invasion ability was measured by matrigel invasion assays. Quantification was performed by counting the stained PANC-1 and ASPC-1 cells that invaded to the lower chamber under a light microscopy. All the results were reproducible in three independent experiments. **. P<0.01 as compared to the control.

The effect of miR-130b on the pancreatic cancer cell invasiveness was investigated by Matrigel invasion assays. We found that the number of invading PANC-1 cells transfected with the miR-130b mimics (57±5 cells/HP; HP: high power magnification field) was remarkably lower than the number of those transfected with miR-NC (122±9 cells/HP; Fig. 4A). Similar results were also obtained for the invasiveness of ASPC-1 cells transfected with the miR-130b mimics (Fig. 4B). It revealed that the miR-130b mediated the pancreatic cancer cell invasiveness.

### STAT3 could be the Direct Target of miR-130b

**Figure 5:**
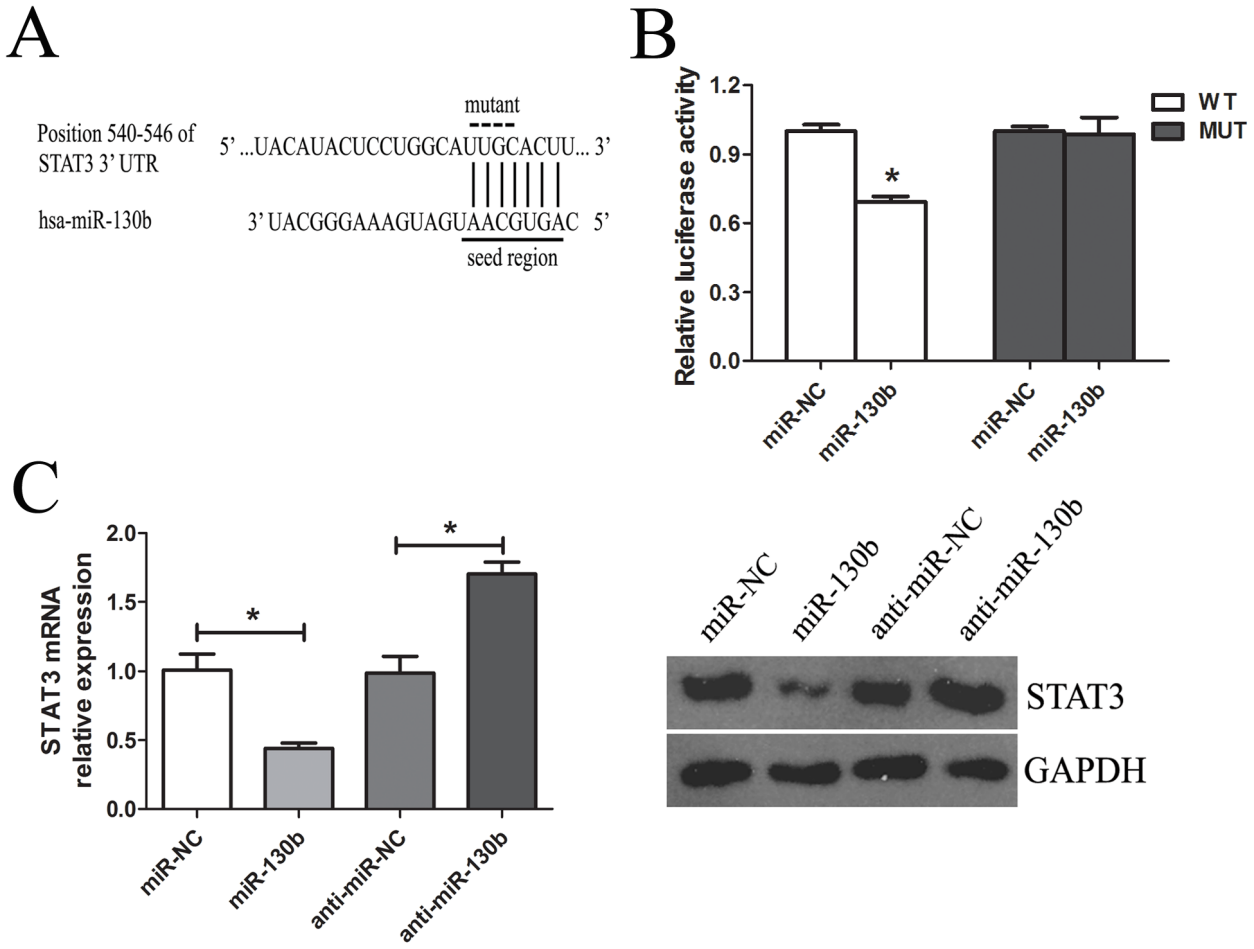
STAT3 is the direct target of miR-130b in PANC-1 cells. (**A**) (Top panel) The human STAT3 3′UTR fragment containing wild-type or mutated miR-130b–binding sequence. (Bottom panel) The miR-130b and the miR-130b-binding site in the 3′UTR of STAT3. (**B**) Luciferase reporter assay with cotransfection of wild-type or mutant 3′UTR (100 ng) and miR-130b or miR-NC (50 nM) in PANC-1 cells. Firefly luciferase activity of each sample was normalized against Renilla luciferase activity. (**C**) The effects of miR-130b or anti-miR-130b on the expression of endogenous STAT3. QRT-PCR (left panel) and western blot (right panel) were used for monitoring the STAT3 expression in PANC-1 cells 48 h after the transfection with miR-130b or anti-miR-130b (50 nM). All data from three separate experiments are presented as mean±SD. *. P<0.05; **. P<0.01.

To explore the potential target through which the miR-130b inhibits the proliferation and invasion of pancreatic cancer cells, we took advantage of bioinformatics to predict the putative miR-130b targets by using TargetScan, miRanda and PicTar algorithms. Our analysis identified STAT3, a key oncogene, as one of the potential targets for miR-130b. Furthermore, the target sequences of STAT3 3′UTR (wild type, WT) or the mutant sequence (mutant type, MUT) were cloned into the luciferase reporter vector, respectively (Fig. 5A). Our results showed that the miR-130b significantly decreased the firefly luciferase activity in the reporter with wild type 3′UTR, but the activity of mutant 3′UTR vector remained unaffected (Fig. 5B). The PANC-1 cells were further transfected with miR-130b and were examined for STAT3 expression by qRT-PCR and western blot. As shown in Fig. 5C, the miR-130b transfection led to an obvious decrease in STAT3 mRNA and protein expression. On the contrary, transfection of anti-miR-130b resulted in an upregulation in the STAT3 expression.

### STAT3 was Upregulated in Pancreatic Tissues and was Inversely Correlated with miR-130b Levels

**Figure 6:**
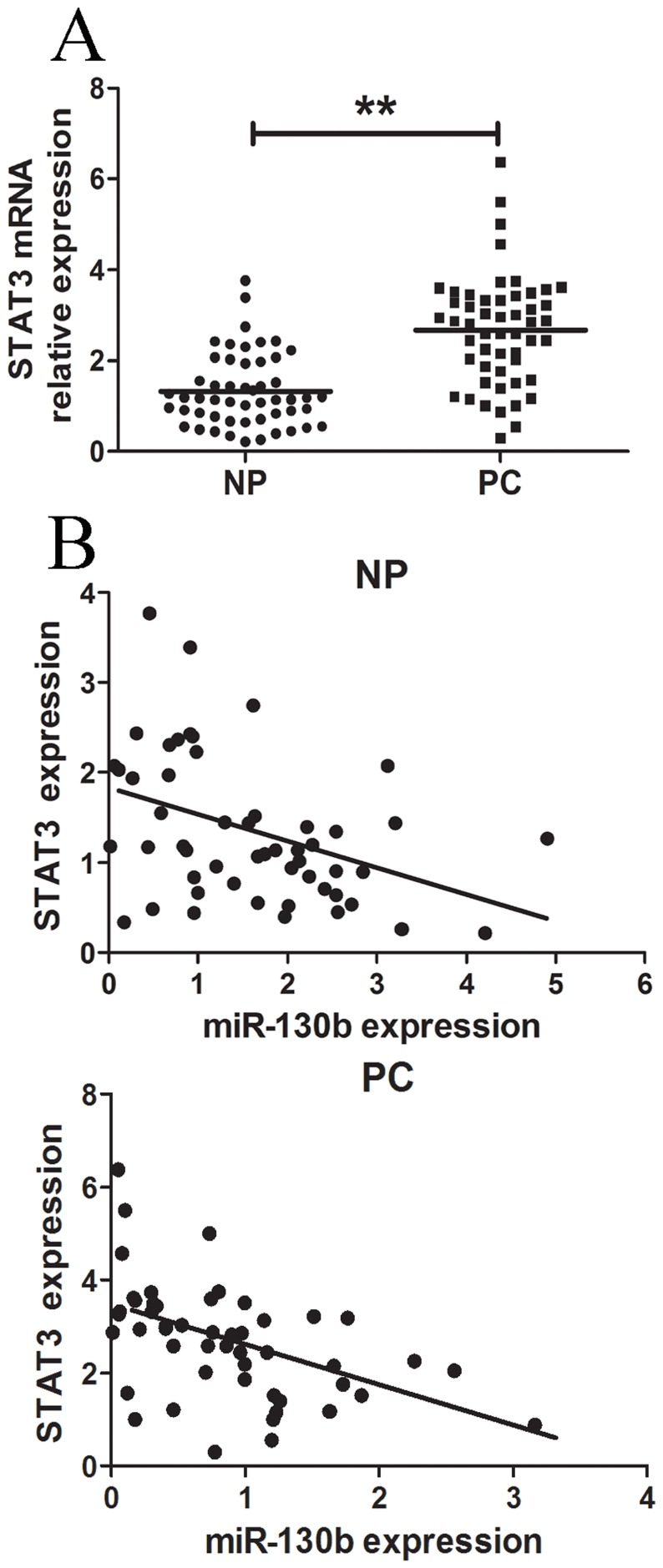
STAT3 was inversely correlated with the miR-130b levels in pancreatic cancer tissues. (A) STAT3 was detected by qRT-PCR in 52 pancreatic cancer (PC) tissues and was matched the adjacent noncancerous pancreatic tissues (NP). The STAT3 abundance was normalized against GAPDH. (B) The relationship between STAT3 and miR-130b expression was explored by Spearman’s correlation in 52 paired PC tissues and adjacent NP tissues. All data from three separate experiments are presented as mean±SD. *. P<0.05; **. P<0.01.

The STAT3 mRNA levels were measured in PC tissues and adjacent noncancerous tissues. As shown in Fig. 6A, the average level of STAT3 expression was significantly higher in PC tissues as compared to that of the NP tissues (P<0.01). A significant inverse correlation was observed between STAT3 and miR-130b expressions in pancreatic cancer tissues (Spearman’s correlation, r = −0.4903; P<0.01) and adjacent noncancerous tissues (r = −0.4026; P<0.001) (Fig. 6B).

## Discussion

The miRNA profiles have been established for a variety of solid and hematologic malignancies [29], [30]. In the recent years, the discovery of miRNAs has undergone significant advances in the understanding of cancer biology by providing additional mechanisms for genetic deregulation. However, little is known about the molecular mechanisms by which of miRNAs modulation in the process of tumorigenesis and the behavior of pancreatic cancer cells. In this study, we found that miR-130b was frequently down-regulated in both pancreatic cancer tissues and cell lines. It was also revealed that miR-130b suppressed the pancreatic cancer cells proliferation both *in vitro* and *in vivo* by induction of apoptosis and cell cycle arrest, as well as inhibited invasiveness *in vitro*. Furthermore, also STAT3 was characterized as a functional target for miR-130b.

MiRNAs have been reported to be important in pancreatic cancers, while the associations with survival and clinical outcomes are largely unclear. The survival analysis of present study revealed that miR-130b deregulation correlated with shorter survival time of pancreatic cancer patients. Meanwhile, the clinical data showed that miR-130b deregulation was significantly associated with large tumor size, late TNM stage, lymphatic invasion and distant metastasis. In addition, multivariate analysis indicated that the low miR-130b expression, advanced TNM stage and distant metastasis were significant prognostic factors for a poor overall survival rate of pancreatic cancer patients. Thus, we propose that our observations provide insights to the importance of miR-130b that could be a novel biomarker to predict the prognosis and progression of patients with pancreatic cancer.

Recently, modulation of miRNAs is believed to hold substantial clinical potential in cancer therapy [31]. Yip et al. revealed that the downregulated miR-130b was highly correlated with the aggressive phenotype of papillary thyroid carcinoma [18]. Yang et al. showed that the miR-130b downregulation promoted the development of multidrug resistant ovarian cancer partially by targeting the 3′-UTR of CSF-1, while miR-130b silencing might be mediated by DNA methylation [32]. However, the other results demonstrated that miR-130b was significantly upregulated and acted as a tumor promoter. Lai et al. demonstrated that miR-130b was obviously overexpressed in gastric cancer and its overexpression increased cell viability and reduced cell death [13]. Liu et al. showed that miR-130b was overexpressed in hepatic cell cancer and might be a serum biomarker with clinical values for hepatic cell cancer screening [33]. Since the effect of miR-130b in pancreatic cancer was far from defined, this study aimed to identify the function of miR-130b in pancreatic cancer. The restoration of miR-130b was found to significantly suppress the proliferation of PANC-1 and ASPC-1 cells by induction of apoptosis and cell cycle arrest at S phase. Furthermore, the growth of xenograft tumors was significantly inhibited after being transfected with LV-miR-130b. These results implied that miR-130b might act as an inhibitor in the progress of pancreatic carcinogenesis. Furthermore, these results showed heterogeneity in the function of miR-130b that was dependent on the cancer type and cellular context.

Invasion and metastasis were the major causes of bad prognosis in patients with pancreatic cancer. Many studies have shown that there was a close correlation between invasion/metastasis of pancreatic cancer and miRNAs, such as miR-20 and miR-146a [9], [34]. Therefore, unraveling the complex role of miRNAs in the process of pancreatic cancer metastasis might provide new insights for some therapeutic consequences. Our clinical results showed that miR-130b was significantly decreased in the patients with invasion/metastasis. Moreover, patients with a higher expression of miR-130b were less frequently associated with invasion/metastasis. We further identified the role of miR-130b in pancreatic cancer cell invasiveness. After restoration of miR-130b, the invasiveness of PANC-1 and ASPC-1 cells was clearly inhibited. These results intensively implied that miR-130b could be involved in the pancreatic cancer metastasis progression. Therefore, approaches to introduce miR-130b into cancer cells might be potentially feasible for clinical treatment of pancreatic cancer, especially for those with lower miR-130b expression in tumor tissues.

STAT3, a member of the signal transduction and activation of transcription (STAT) family, has been frequently overexpressed in a wide variety of human tumors including pancreatic cancer [35]–[37]. STAT3 participates in the initiation and development of cancers by promoting cell proliferation, inhibiting cell apoptosis, promoting angiogenesis, invasion and metastasis [38], [39]. In this study, an important molecular association between miR-130b and STAT3 was demonstrated. The bioinformatics analysis indicated that STAT3 might be one of the potential targets for miR-130b. The luciferase activity data showed that miR-130b was able to directly target the 3′UTR of STAT3. The qRT-PCR and western blot results showed that the overexpression of miR-130b downregulated the expression of STAT3 in pancreatic cancer cells by both interfering with and degrading mRNA, whereas the anti-miR-130b upregulated the STAT3 expression. Finally, the inverse correlation between miR-130b and STAT3 expression in PC and NP tissues was further confirmed that miR-130b downregulation resulted in STAT3 overexpression. Taken together, these data demonstrated that miR-130b could inhibit cell growth and invasion of pancreatic cancer by targeting STAT3.

In summary, our present study demonstrated that the deregulated expression of miR-130b was associated with poor prognosis and aggressive phenotype of pancreatic cancer. This study also implied that miR-130b played an important role in the regulation of pancreatic cancer malignant behavior including cell proliferation and invasion by directly targeting STAT3. In general, these results indicated that miR-130b might be applied as a potential prognostic biomarker and inhibitor in pancreatic cancer.

## Supporting Information

Table S1 Sequences of qRT-PCR primers. ^a^ Forward primer. ^b ^Reverse primer.
(DOCX)

Table S2 Sequences of RNA oligonucleotides.
(DOCX)
