# Correction: MiR-130b Is a Prognostic Marker and Inhibits Cell Proliferation and Invasion in Pancreatic Cancer through Targeting STAT3

**DOI:** 10.1371/annotation/497d9208-190a-44b7-ac27-aea12183f47c

**Published:** 2013-09-23

**Authors:** Gang Zhao, Jun-gang Zhang, Ying Shi, Qi Qin, Yang Liu, Bo Wang, Kui Tian, Shi-chang Deng, Xiang Li, Shuai Zhu, Qiong Gong, Yi Niu, Chun-you Wang

There was information omitted from the Funding section. The correct version of the Funding is available below.

This study was supported by grants from the National Science Foundation Committee (NSFC) of China (grant number: 30972900 and600 30600594), and the Research Special Fund for Public Welfare Industry of Health of China (grant number: 201202007). The funders had no role in study design, data collection and analysis, decision to publish, or preparation of the manuscript. 

