# Supplementary material for: MiR-130b Is a Prognostic Marker and Inhibits Cell Proliferation and Invasion in Pancreatic Cancer through Targeting STAT3
Source: PLoS One. 2013 Sep 10;8(9):e73803. doi: 10.1371/journal.pone.0073803 (PMC3769379; doi:10.1371/journal.pone.0073803)
Supplement: Table S1 — Sequences of qRT-PCR primers. a Forward primer. b Reverse primer. (DOCX) [file pone.0073803.s001.docx]

**Table S1. Sequences of qRT-PCR primers**

| Primer | Primer Sequence |
| --- | --- |
| STAT3-F^a^ | 5’-GCACAGATTGCCTGCATTG-3’ |
| STAT3-R^b^ | 5’-CTGCTAATGACGTTATCCAGT-3’ |
| GAPDH-F^a^ | 5’-GAAGGTGAAGGTCGGAGTC-3’ |
| GAPDH-R^b^ | 5’-GAAGATGGTGATGGGATT-3’ |
| miR-130b-F^a^ | 5’-CAGTGCAATGATGAAAGGGCAT-3’ |
| U6-F^a^ | 5’-CTCGCTTCGGCAGCACA-3’ |

^a^ Forward primer

^b^ Reverse primer
