# Supplementary material for: MiR-130b Is a Prognostic Marker and Inhibits Cell Proliferation and Invasion in Pancreatic Cancer through Targeting STAT3
Source: PLoS One. 2013 Sep 10;8(9):e73803. doi: 10.1371/journal.pone.0073803 (PMC3769379; doi:10.1371/journal.pone.0073803)
Supplement: Table S2 — Sequences of RNA oligonucleotides. (DOCX) [file pone.0073803.s002.docx]

**Table S2. Sequences of RNA oligonucleotides**

|  | Sequence (5’-3’) |
| --- | --- |
| hsa-miR-130b mimics | CAGUGCAAUGAUGAAAGGGCAU |
| miR-NC | CGGUGAUAAUCUUUGUAGACG |
| anti-miR-130b | AUGCCCUUUCAUCAUUGCACUG |
| Inh-NC | CAGUACUUUUGUGUAGUACAA |
